# How well do ICD-9 physician claim diagnostic codes identify confirmed pertussis cases in Alberta, Canada? A Canadian Immunization Research Network (CIRN) Study

**DOI:** 10.1186/s12913-017-2321-1

**Published:** 2017-07-12

**Authors:** Sumana Fathima, Kimberley A. Simmonds, Steven J. Drews, Lawrence W. Svenson, Jeffrey C. Kwong, Salaheddin M. Mahmud, Susan Quach, Caitlin Johnson, Kevin L. Schwartz, Natasha S. Crowcroft, Margaret L. Russell, For the Canadian Immunization Research Network (CIRN) Provincial Collaborative Network Vaccine Effectiveness Working Group, Natasha Crowcroft, Jeffrey C. Kwong, Margaret L. Russell, Lawrence W. Svenson, Steven J. Drews, Kimberley A. Simmonds, Salaheddin M. Mahmud, Shelley Deeks, Frances Jamieson, Sarah Wilson

**Affiliations:** 1 Cumming School of Medicine, University of Calgary, Calgary, Alberta Canada; 2 Alberta Ministry of Health, Edmonton, Alberta Canada; 3 Provincial Laboratory (ProvLab) for Public Health, Edmonton, Alberta Canada; 4 University of Alberta, Edmonton, Alberta Canada; 5 University of Toronto, Toronto, Canada; 6 Vaccine and Drug Evaluation Centre, University of Manitoba, Winnipeg, MB Canada; 7 Public Health Ontario, Toronto, Canada; 8 The Institute for Clinical Evaluative Sciences, Toronto, ON Canada; 9 The Hospital for Sick Children, Toronto, ON Canada; 10 Department of Community Health Sciences, University of Calgary, 3rd Floor TRW, 3280 Hospital Drive NW, Calgary, AB T2N 4Z6 Canada

**Keywords:** Pertussis, Validation studies, International classification of diseases, ICD 9, Sensitivity, Specificity, Health services research

## Abstract

**Background:**

Rates of *Bordetella pertussis* have been increasing in Alberta, Canada despite vaccination programs. Waning immunity from existing acellular component vaccines may be contributing to this. Vaccine effectiveness can be estimated using a variety of data sources including diagnostic codes from physician billing claims, public health records, reportable disease and laboratory databases. We sought to determine if diagnostic codes from billing claims (administrative data) are adequately sensitive and specific to identify pertussis cases among patients who had undergone disease-specific laboratory testing.

**Methods:**

Data were extracted for 2004–2014 from a public health communicable disease database that contained data on patients under investigation for *B. pertussis* (both those who had laboratory tests and those who were epidemiologically linked to laboratory-confirmed cases) in Alberta, Canada. These were deterministically linked using a unique lifetime person identifier to the provincial billing claims database, which contains International Classification of Disease version 9 (ICD-9) diagnostic codes for physician visits. We examined visits within 90 days of laboratory testing. ICD-9 codes 033 (whooping cough), 033.0 (*Bordetella pertussis*), 033.1 (*B. parapertussis*), 033.8 (whooping cough, other specified organism), and 033.9 (whooping cough, other unspecified organism) in any of the three diagnostic fields for a claim were classified as being pertussis-specific codes. We calculated sensitivity, specificity, positive (PPV) and negative (NPV) predictive values.

**Results:**

We identified 22,883 unique patients under investigation for *B. pertussis*. Of these, 22,095 underwent laboratory testing. Among those who had a laboratory test, 2360 tested positive for pertussis. The sensitivity of a pertussis-specific ICD-9 code for identifying a laboratory-confirmed case was 38.6%, specificity was 76.9%, PPV was 16.0%, and NPV was 91.6%.

**Conclusion:**

ICD-9 codes from physician billing claims data have low sensitivity and moderate specificity to identify laboratory-confirmed pertussis among persons tested for pertussis.

**Electronic supplementary material:**

The online version of this article (doi:10.1186/s12913-017-2321-1) contains supplementary material, which is available to authorized users.

## Background

Publicly funded vaccination programs in infants and adolescents, improved diagnostic testing and surveillance have resulted in declines in pertussis in Canada. However, resurgence beyond that expected for disease cyclicity has occurred recently [1]. This problem is not confined to Canada [2] One of the many possible explanations is that vaccine effectiveness may wane over time [3–5].

In Canada, acellular pertussis vaccines replaced whole cell pertussis vaccines in 1997 [6]. Recent disease resurgence may be associated with a change from whole cell to acellular pertussis vaccines, and further complicated in that the duration of protection after receipt of a booster dose of acellular vaccine might be shorter among persons primed with acellular rather than whole cell vaccine [7]. The comparative effectiveness and duration of protection of pertussis vaccination among populations primed and boosted with whole cell versus acellular pertussis vaccines warrant study. Such studies may employ a test-negative case-control design [8] or other design [9], and may use a variety of data sources. As administrative data can be a potential data source for such endeavors that is readily available, inexpensive, efficient, and powerful, we wanted to gain a better understanding of the accuracy of these data for case identification.

We investigated the validity of ICD-9 diagnostic codes submitted as part of physician billing claims using the current Alberta case definition for pertussis as the reference standard. This reference standard defines a confirmed case as one that is laboratory confirmed or epidemiologically linked to a laboratory-confirmed case [10]. Our focus is on those reference standard cases that are laboratory confirmed.

## Methods

Alberta, with a population of 4.1 m [10], is the fourth largest province in Canada and like all Canadian provinces has a universal publicly funded health care system that covers over 99% of the population [11]. In this system, physicians are paid for services provided to patients (hospitalized patients as well as out-patients) by the Ministry of Health. All Albertans are assigned a Personal Health Number that is a unique lifetime identifier (ULI). Physicians are also assigned a unique physician identification code to support the submission of claims. A physician claim for payment must, in addition to a billing code for the service provided, include the patient ULI and up to three ICD-9 diagnostic codes for the health condition (s) for which the service was provided. Most physicians are paid on a fee-for-service basis [12], with those being paid through non fee-for-service plans (alternative payment plans) also being required to ‘shadow bill’ [13]. ‘Shadow billing’ is a process where non fee-for-service physicians must submit information for services provided as if they were submitting a fee-for-service claim, even though physician payment is not directly linked to the services reported [14]. Claims data have face validity [13] and preliminary assessment of the accuracy of dignostic recording for claims that are shadow billed has been shown to be similar to that of claims submitted by fee-for-service physicians [15]. The Supplemental Enhanced Service Event (SESE) database contains the ICD-9 diagnostic codes submitted as part of physician claims for payment [11].

Since 2004, the Provincial Laboratory for Public Health (ProvLab) is the only laboratory in Alberta to perform pertussis testing. At the same time, polymerase chain reaction (PCR) testing replaced direct fluorescent antibody testing and/or culture for pertussis. Specimens are tested for pertussis only if pertussis testing is specifically ordered, as specimens require special transport medium. The ProvLab’s Data Integration for Alberta Laboratories (DIAL) system, described elsewhere [16], was used to extract the final classification information for specimens (positive or negative) tested for pertussis from the ProvLab Laboratory Information System.

The Alberta Communicable Disease Reporting System (CDRS) contains information on all communicable diseases (including pertussis) that are notifiable by law [17].

We identified all persons for whom a laboratory specimen had been submitted for pertussis testing. Records were deterministically linked across databases (using the patient ULI) to assess the relationship between pertussis cases identified by ICD-9 diagnostic codes from physician claims records, and confirmed cases according to CDRS. We calculated the sensitivity, specificity, positive predictive value (PPV) and negative predictive value (NPV) of ICD-9 codes for a physician diagnosis of pertussis during the period 2004 to 2014 [18]. We describe the distribution of ICD-9 codes for claims for each of true positives, false positives, false negatives, and true negatives. We explored the relationship between physician-diagnosed cases of pertussis and CDRS-confirmed cases (i.e., we did not link to the ProvLab database). For this analysis, we included all CDRS-confirmed cases whether or not a laboratory test had been done.

Incident cases of pertussis (the earliest dated CDRS classification as a confirmed case) were extracted from CDRS for the period January 1, 2004–December 31, 2014. SESE was used to extract all physician claims (in the 90 days prior to CDRS notification) for CDRS-confirmed pertussis cases using *B. pertussis*-specific ICD-9 codes [033 (whooping cough), 033.0 (*Bordetella pertussis*), 033.1 (*B. parapertussis*), 033.8 (whooping cough, other specified organism), 033.9 (whooping cough, other unspecified organism)] in any of the three diagnostic fields for a claim. We calculated the sensitivity and positive predictive value of the physician diagnosis of pertussis [18].

### Ethics

The study was approved by the University of Calgary Conjoint Health Research Ethics Board (REB 15–0732) and by the University of Alberta Health Research Ethics Board, Health Panel Pro00057348. Written informed consent from individual patients was not required because data extraction and linkages were performed by Alberta Ministry of Health personnel (data custodian for all databases used in this study) and data were de-identified prior to release for this study.

### Definitions

The Alberta Public Health case definition (CDRS) defines a confirmed case of pertussis as

- Isolation of *Bordetella pertussis* from an appropriate clinical specimen (e.g., nasopharyngeal swab) **OR**
- Detection of *B. pertussis* nucleic acid (e.g., PCR) from an appropriate clinical specimen **AND** compatible cough illness **OR**
- a person who is epidemiologically linked to a laboratory-confirmed case with compatible cough illness for which there is no other known cause [10].

Physician-diagnosed cases of pertussis are those for which, in any of the three fields for diagnostic codes in a claim, a *B. pertussis*-specific ICD-9 code [033 (whooping cough), 033.0 (*Bordetella pertussis*), 033.1 (*B. parapertussis*), 033.8 (whooping cough, other specified organism), or 033.9 (whooping cough, other unspecified organism)] was submitted.

We defined true positives (TP) as physician-diagnosed cases that also met the CDRS case definition of confirmed case and for which a laboratory test for pertussis had been submitted and classified as positive. False negative (FN) cases were those for which a laboratory test had been submitted and been classified as positive for pertussis, that also met the CDRS case definition of confirmed pertussis, but for which physician claims did not include a pertussis-specific ICD-9 code. False positive (FP) cases were those for whom physician claims had one or more pertussis-specific code but were not confirmed cases by the CDRS case definition and for which a laboratory test was negative. We defined a true negative case (TN) as one for which a laboratory test for pertussis was submitted to ProvLab and tested negative and did not meet the CDRS case definition for a confirmed case of pertussis, and for whom the physician claim did not include any pertussis-specific ICD-9 codes.

For our exploration of physician-diagnosed cases of pertussis and CDRS-confirmed cases, we defined a true positive as being positive according to both CDRS classification as a confirmed case and physician diagnosis of pertussis. False positive (FP) cases were those for whom physician claims had one or more pertussis-specific code but were not confirmed cases by the CDRS case definition.

Statistical analysis was done using SPSS Version 22 (IBM SPSS Inc. Chicago, IL).

## Results

A total of 22,883 patients were identified after linking CDRS with SESE, of whom 22,095 had a laboratory test for pertussis ordered. Of the 3148 confirmed pertussis cases in CDRS, 2360 were laboratory confirmed (75%) while 788 were epidemiologically linked without laboratory testing. ICD-9 diagnostic codes were missing from physician claims for 100 patients, thus there were 21,995 records with ICD codes among the patients who had undergone laboratory testing.

Among the 2360 laboratory-confirmed cases of pertussis, the hospitalization status was unknown for 76 (3.2%). There were 215/2360 (9.1%) that were hospitalized. Most of the hospitalized cases (153/215, 71.1%) were aged less than one year. However, only 181 of the 2069 not hospitalized cases (8.7%) were in this age group.

Among patients for whom a laboratory test for pertussis was ordered, the sensitivity of a physician claim diagnosis specific for pertussis was 38.6% (95% CI: 36.6–40.6%) and the specificity 76.9% (95% CI: 76.3–77.5%). The positive and negative predictive values (respectively) were 16.0% (95% CI: 15.0–17.0%) and 91.6% (95% CI: 91.2–92.0%) [Table 1]. Table 2 displays the frequency of the ICD-9 codes from the physician claims for persons who had a laboratory test for pertussis for TP, FP, FN and TN cases. Physician claim diagnostic codes covered the 17 chapters of ICD-9. Among the FN, 100 did not have diagnostic codes; the rest used codes from Chapter VIII (Diseases of the respiratory system) and the large majority of these were coded 460–466 (Acute respiratory infections). Among the TN cases, more than 10% of the submitted codes were also coded under Chapter VIII (21.4%), also most commonly as 460–466, Acute respiratory infections. The next most commonly used ICD-9 chapter for TN claims was XVI (Symptoms, signs and ill-defined conditions) followed by Chapter XV (Certain conditions originating in the perinatal period) at 14 and 12.8%, respectively. A small proportion of TN cases (4.3%) had codes from ICD-9 Chapter I (Infectious and parasitic diseases).

**Table 1:** Performance measures: physician billing ICD-9 codes (claims data) vs. laboratory confirmed pertussis cases (CDRS) 2004–2014

Billing codes from physician claims^a^	Laboratory confirmed pertussis case^b^ (CDRS)		
	Yes	No	Total
Pertussis specific ICD-9 code^c^	872 (TP)	4563 (FP)	5435
Non-pertussis ICD-9 code	1388 (FN)	15172 (TN)	16560
Total	2260	19735	21995
Sensitivity = 38.6% (95% CI: 36.6%–40.6%); Specificity = 76.9% (95% CI: 76.3%–77.5%). Positive predictive value = 16.0% (95% CI: 15.1%–17.0%); Negative predictive value = 91.6% (95% CI: 91.2%–92.0%).			

*TP* true positive, *FP* false positive, *FN* false negative, *TN* true negative, *CI* confidence interval

^a^Test measurement

^b^Reference standard

^c^033 (whooping cough), 033.0 (*Bordetella pertussis*), 033.1 (*B. parapertussis*), 033.8 (whooping cough, other specified organism), 033.9 (whooping cough, other unspecified organism)

**Table 2:** Frequencies of ICD-9 chapters & specific diagnostic codes from the physician claims among persons who had a laboratory test for pertussis by case status

ICD 9 CHAPTER	ICD-9 CODES	CASE STATUS			
		TP (%)	FP (%)	FN (%)	TN (%)
I. Infectious and parasitic diseases	001–139	872 (100)	4563 (100)	0	656 (4.3)
	033 (whooping cough)	747 (86.0)	0	0	21 (0.1)
	033.0 (*B. pertussis*)	0	3949 (87.0)	0	0
	033.1 (*B. parapertussis*)	12 (1.4)	56 (1.3)	0	0
	033.8 (Whooping cough, other specified organism)	2 (0.2)	34 (0.8)	0	0
	033.9 (Whooping cough, other unspecified organism)	111 (12.7)	524 (11.4)	0	2 (>0.1)
	Other codes (001–032.9; 034–139)	0	0	0	633 (4.2)
II. Neoplasms	140–239	0	0	0	110 (0.7)
III. Endocrine, nutritional and metabolic diseases and immunity disorders	240–279	0	0	0	299 (2.0)
IV. Diseases of blood and blood-forming organs	280–289	0	0	0	38 (0.2)
V. Mental disorders	290–319	0	0	0	614 (4.0)
VI. Diseases of the nervous system and sense organs	320–389	0	0	0	1226 (8.0)
VII. Diseases of the circulatory system	390–459	0	0	0	255 (1.7)
VIII. Diseases of the respiratory system	460–519	0	0	1388 (100)	3256 (21.4)
	460–466 (Acute respiratory infections)	0	0	1061 (76.4)	2550 (16.8)
	470–479 (Other diseases of upper respiratory tract)	0	0	41 (3.0)	113 (0.7)
	480–487 (Pneumonia and influenza)	0	0	118 (8.5)	139 (0.9)
	490–496 (Chronic obstructive pulmonary disease and allied conditions)	0	0	159 (11.5)	412 (2.7)
	500–508 (Pneumoconiosis and other lung diseases due to external agents)	0	0	0	3 (>0.1)
	510–519 (Other disease of respiratory system)	0	0	9 (0.6)	39 (0.2)
IX. Diseases of the digestive system	520–579	0	0	0	374 (2.4)
X. Diseases of the genitourinary system	580–629	0	0	0	675 (4.4)
XI. Complications of pregnancy, childbirth and the puerperium	630–676	0	0	0	190 (1.2)
XII. Diseases of the skin and subcutaneous tissue	680–709	0	0	0	722 (4.7)
XIII. Diseases of the musculoskeletal system and connective tissue	710–739	0	0	0	1095 (7.2)
XIV. Congenital anomalies	740–759	0	0	0	221 (1.4)
XV. Certain conditions originating in the perinatal period	760–779	0	0	0	1951 (12.8)
XVI. Symptoms, signs and ill-defined conditions	780–799	0	0	0	2130 (14.0)
XVII. Injury and poisoning	800–999	0	0	0	1360 (9.0)
	Total	872	4563	1388	15172

*TP* True Positive (physician diagnosed case that was also laboratory test positive for pertussis), *FP* False Positive (Physician diagnosed case that was laboratory test negative for pertussis), *FN* False Negative (Physician did not diagnose pertussis, but laboratory test positive for pertussis), *TN* True Negative (physician did not diagnose pertussis, laboratory test negative for pertussis)

When the reference standard of a confirmed case of pertussis according to the CDRS case definition (which includes cases that did not have a laboratory test) was used (Additional file 1), the sensitivity of a physician claims diagnosis specific to pertussis declined to 32.6% (95% CI: 30.9–34.1%), while the positive predictive value increased to 18.3% (95% CI: 17.3–19:4%). A similar proportion of these cases were hospitalized (7%) as those cases that had a laboratory test (9.1%). As was the case for those cases that had a laboratory test, most of the CDRS-confirmed cases were also less than one year of age (69.5%).

## Discussion

We found that, for all patients for whom a laboratory test for pertussis was submitted, the sensitivity of physician claims diagnostic code was low (38.6%) although the specificity was moderate (76.9%). This was mirrored in the corresponding positive and negative predictive values of 16.0 and 91.6% respectively. When the reference standard for a confirmed case was taken to be the CDRS definition inclusive of cases that were epidemiologically linked, but which had not been subjected to laboratory testing, the sensitivity of physician claim diagnosis declined somewhat to 32.6% while the PPV increased to 18.3%. This latter is not surprising as PPV is influenced by the prevalence of a condition and the number of reference standard cases of pertussis increased. We used the diagnostic code from only one claim per person; it is possible that requiring more than one visit (claim) with an appropriate ICD code may increase specificity.

We found a low sensitivity but a relatively higher specificity to identify pertussis cases by using ICD-9 codes from physician billing claims. The specificity of ICD-9 codes was an indicator of the proportion of cases that were coded as other respiratory diseases by physicians and never reported to public health. We cannot estimate the number of true negative cases that were not tested. If these had been included, specificity and negative predictive value would have both been higher, but sensitivity and positive predictive value would have been unaffected. The number of false positives could be due to many reasons. The use of a single diagnostic code could be part of describing symptoms, interpreting a diagnostic test, a confirmed diagnosis, or an error in coding [19]. The relatively higher specificity could be useful for other studies or diseases where there is a need to identify non-cases [20]. Unless a rare or emerging disease is of threat, a highly specific test is important to reduce the numbers of false positives [21] and to produce higher diagnostic accuracy of obtaining a negative result. Therefore, ICD-9 claims codes might be used for diseases where obtaining false positives is an issue. Although ProvLab has a highly sensitive real-time PCR assay for pertussis [16], it can produce negative results (due to low DNA, inadequate sampling, degradation); false negative cases are not sent to public health and are thus not in CDRS. As not all cases that are considered to be true cases according to the Alberta case definition (which includes persons who are “epidemiologically linked to a laboratory-confirmed case with compatible cough illness for which there is no other known cause”) are laboratory tested, the number of true positives is also underestimated. Even with low sensitivity, Alberta’s robust laboratory and public health system can produce linked cases to study adverse effects, immunization coverage and vaccine effectiveness [20]. For a test-negative case-control design to study vaccine effectiveness, a combination of data sources such as laboratory confirmed cases, disease notifications, and immunization records are a better approach than using any single data source alone.

Our analyses included both hospitalized and non-hospitalized cases. Few studies have compared the accuracy of ICD diagnostic codes for outpatients with those of hospitalized patients. Watkins and colleagues [22] examined physician billing diagnostic codes (assigned at the time of the visit by the medical provider) with inpatient diagnostic codes for five enteric diseases. They found that a much larger proportion of the inpatient (about 50%) compared to only 7% of the outpatient laboratory confirmed cases were identified by the ICD- 9 codes. However, the inpatient codes were assigned at the time of discharge, when the results of laboratory testing were known, in contrast to the outpatient codes. It has been suggested that broad syndrome-based ICD-9 codes, rather than specific ICD–9 diagnostic codes be used to identify diseases with complex case-definitions, particularly in the outpatient setting [23]; however the addition of laboratory testing results may substantively improve the PPV of cases identified by ICD-9 code for relatively common communicable diseases. Others have suggested that physician claims can be used for syndromic surveillance when administrative data are sufficiently timely for the purposes of surveillance [24, 25]. Using ICD codes alone can be one way to track trends in pertussis activity if laboratory confirmation is not available. We posit that the most appropriate method of case identification may depend upon the purpose of a specific study: research, population health assessment or public health surveillance. Furthermore the purposes of a surveillance system are many [26]. For example, surveillance of vaccine effectiveness requires high specificity while surveillance for burden of disease requires high sensitivity. Thus the relative importance of sensitivity vs. specificity may also depend upon the purpose of the surveillance.

Our study has several strengths with respect to novelty as well as to the data sources used to validate claims. Although many validation studies of administrative data have been published, very few have addressed outpatient data rather than hospital data. Further, most validation studies address codes for non-infectious diseases. A few papers have been published that have validated physician billing codes for immunization [20, 27]. A small number of published articles have validated codes for diagnoses of acute respiratory infections in primary care [28–30]. However, none of these investigators studied codes for pertussis. Further, each health care and public health system in each province of Canada is unique. This is the first study that has addressed this issue in Alberta.

A recent study in Alberta concluded that diagnosis using ICD-9 codes via physician claims has a high validity and that such data can be valuable for health care planning and surveillance. Because of universal health care in Canada, a very large proportion of health care visits are captured in administrative settings such as physician claims [13]. In our jurisdiction ProvLab performs the majority of tests for many notifiable diseases and since 2004 performs all pertussis testing for the province. Thus the ProvLab laboratory information system is a rich and reliable data source to obtain laboratory testing data, particularly for diseases for which laboratory reporting is mandatory as is the case for pertussis [17].

## Conclusion

Physician billing ICD-9 codes have low sensitivity, and billing codes alone are not the most appropriate measure to identify pertussis. For a disease like pertussis, laboratory diagnosis is not only essential for understanding the true burden of this disease, but is a necessary component for prospective studies that might examine vaccine effectiveness and waning immunity.

## Additional file

Additional file 1: Table S1. Performance measures for physician billing codes compared to the CDRS case definition for confirmed cases that includes both laboratory confirmed cases and those cases that were not laboratory tested but which were epidemiologically linked to a laboratory confirmed case (2004–2014). The performance measures are sensitivity, specificity, positive and negative predictive values. (DOCX 15 kb)

## Abbreviations

CDRS: Communicable disease reporting system
CI: Confidence interval
DIAL: Data integration for Alberta laboratories
FN: False negative
FP: False positive
ICD-9: International classification of disease version 9
NPV: Negative predictive value
PCR: Polymerase chain reaction
PPV: Positive predictive value
ProvLab: Provincial laboratory for public health
SESE: Supplemental enhanced service event
TN: True negative
TP: True positive
ULI: Unique lifetime identifier

## References

[1] Smith T, Rotondo J, Desai S, Deehan H (2014). Pertussis surveillance in Canada: trends to 2012. Can Commun Dis Rep.

[2] Halperin SA (2014). Pertussis: a global perspective. Can Commun Dis Rep.

[3] Ruderfer D, Krilov LR (2015). Vaccine-preventable outbreaks: still with us after all these years. Pediatr Ann.

[4] Tartof SY, Lewis M, Kenyon C, White K, Osborn A, Liko J (2013). Waning immunity to pertussis following 5 doses of DTaP. Pediatrics.

[5] McGirr A, Fisman DN (2015). Duration of pertussis immunity after DTaP immunization: a meta-analysis. Pediatrics.

[6] Alberta Health. Alberta Immunization Policy Guidelines: Appendix 2 History of biologicals administered in Alberta 2014 [cited 2015 Aug 21]. Available from http://www.health.alberta.ca/documents/AIP-History-Alberta-Biologicals-Introduction.pdf. 6-1-2013.

[7] World Health Organization. Pertussis vaccines: WHO position paper - September 2015. [cited 2015 Aug 30]. Available from: http://www.who.int/wer/2015/wer9035/en/ 90[35], 433–460. 8-28-2015.

[8] Foppa IM, Haber M, Ferdinands JM, Shay DK (2013). The case test-negative design for studies of the effectiveness of influenza vaccine. Vaccine.

[9] World Health Organization. Overview of Vaccine Efficacy and Effectitveness. [cited 2015 Aug 5].Available from http://www.who.int/influenza_vaccines_plan/resources/Session4_VEfficacy_VEffectiveness.PDF.7-30-2015.

[10] Alberta Health. Public Health Notifiable Disease Management Guidelines. [cited 2015 April 11]. Available from: https://open.alberta.ca/publications/pertussis.

[11] Russell ML, Dover DC, Simmonds KA, Svenson LW (2014). Shingles in Alberta: before and after publicly funded varicella vaccination. Vaccine.

[12] Government of Alberta. Alberta Health: Alberta Health Care Insurance Plan Statistical Supplement 2013/2014. Government of Alberta.5-24-2015. 2-2-2016.

[13] Cunningham CT, Cai P, Topps D, Svenson LW, Jetté N, Quan H (2014). Mining rich health data from Canadian physician claims: features and face validity. BMC Res Notes.

[14] Canadian Institute for Health Information. The Status of Alternative Payment Programs for Physicians in Canada 2002.2003 and Preliminary Information for 2003.2004. Canadian Institute for Health Information.2004. 2-2-2016.

[15] Cunningham CT, Jette N, Li B, Dhanoa RR, Hemmelgarn B, Noseworthy T (2015). Effect of physician specialist alternative payment plans on administrative health data in Calgary: a validation study. CMAJ Open.

[16] Fathima S, Ferrato C, Lee BE, Simmonds K, Yan L, Mukhi SN (2014). *Bordetella pertussis* in sporadic and outbreak settings in Alberta, Canada, July 2004-December 2012. BMC Infect Dis.

[17] Alberta Health. Notifiable Disease and Diseases Under Surveillance List.Government of Alberta.2015. 2-2-2016.

[18] Porta M, Greenland S, Last JM (2008). A dictionary of epidemiology.

[19] Koran LM (1975). The reliability of clinical methods, data and judgments (first of two parts). N Engl J Med.

[20] Schwartz KL, Tu K, Wing L, Campitelli MA, Crowcroft NS, Deeks SL (2015). Validation of infant immunization billing codes in administrative data. Hum Vaccin Immunother.

[21] Zeman DH (1997). The “best” diagnostic test. Swine Health Production.

[22] Watkins M, Lapham S, Hoy W (1991). Use of a medical center’s computerized health care database for notifiable disease surveillance. Am J Public Health.

[23] Sickbert-Bennett EE, Weber DJ, Poole C, MacDonald PDM, Maillard JM (2010). Utility of international classification of diseases, ninth revision, clinical modification codes for communicable disease surveillance. Am J Epidemiol.

[24] Cadieux G, Buckeridge DL, Jacques A, Libman M, Dendukuri N, Tamblyn R (2012). Patient, physician, encounter, and billing characteristics predict the accuracy of syndromic surveillance case definitions. BMC Public Health.

[25] Cadieux G, Buckeridge DL, Jacques A, Libman M, Dendukuri N, Tamblyn R (2011). Accuracy of syndrome definitions based on diagnoses in physician claims. BMC Public Health.

[26] Teutsch SM, Lee LM, Teutsch SM, Thacker SB, St. Louis ME (2010). Considerations in planning a surveillance system. Principles & practice of public health surveillance.

[27] Williams WO, Dajda R (1980). Validation of sources of pertussis immunisation data. J Epidemiol Community Health.

[28] Cadieux G, Tamblyn R (2008). Accuracy of physician billing claims for identifying acute respiratory infections in primary care. Health Serv Res.

[29] Linder JA, Bates DW, Williams DH, Connolly MA, Middleton B (2006). Acute infections in primary care: accuracy of electronic diagnoses and electronic antibiotic prescribing. J Am Med Inform Assoc.

[30] Maselli JH, Gonzales R (2001). Measuring antibiotic prescribing practices among ambulatory physicians: accuracy of administrative claims data. J Clin Epidemiol.

