# Supplementary material for: How well do ICD-9 physician claim diagnostic codes identify confirmed pertussis cases in Alberta, Canada? A Canadian Immunization Research Network (CIRN) Study
Source: BMC Health Serv Res. 2017 Jul 12;17:479. doi: 10.1186/s12913-017-2321-1 (PMC5508611; doi:10.1186/s12913-017-2321-1)
Supplement: Additional file 1: Table S1. — Performance measures for physician billing codes compared to the CDRS case definition for confirmed cases that includes both laboratory confirmed cases and those cases that were not laboratory tested but which were epidemiologically linked to a laboratory confirmed case (2004–2014). The performance measures are sensitivity, specificity, positive and negative predictive values. (DOCX 15 kb) [file 12913_2017_2321_MOESM1_ESM.docx]

Additional table 1. Performance measures: physician billing ICD-9 codes (claims data) vs. all confirmed pertussis cases (CDRS case definition of confirmed case: laboratory confirmed OR case that was not laboratory tested that is epidemiologically linked to a laboratory confirmed case) 2004 – 2014.

| Billing codes from physician claims* | CDRS confirmed case (laboratory confirmed OR epidemiologically linked to laboratory confirmed case) | | |
| --- | --- | --- | --- |
|  | **Yes** | **No** | **Total** |
| **Pertussis specific ICD-9 code***** | 1025 | 4563 | 5588 |
| **Non-pertussis ICD-9 code** | 2123 | 15172 | 17295 |
| **Total** | 3148 | 19735 | 22883 |
| Sensitivity = 32.6% (95% CI: 31.0% to 34.2%)  Specificity = 76.9% (95% CI: 76.3% to 77.5%)  Positive predictive value = 18.30% (95% CI: 17.3% to 19.4%)  Negative predictive value = 87.7% (95% CI: 87.2% to 88.2% ) | | | |

*Test measurement
